# SARS-CoV-2 Seroconversion in Response to Infection and Vaccination: a Time Series Local Study in Brazil

**DOI:** 10.1128/spectrum.01026-22

**Published:** 2022-06-30

**Authors:** Luciano F. Huergo, Nigella M. Paula, Ana C. A. Gonçalves, Carlos H. S. Kluge, Paulo H. S. A. Marins, Haxley S. C. Camargo, Thamyres P. Sant’Ana, Lucas R. P. Farias, Juliane D. Aldrighi, Ênio S. Lima, Guiomar T. Jacotenski, Letícia R. Vargas, Gisele Costa, Karin V. Weissheimer, Maria G. Nazário, Kádima N. Teixeira, Marcelo S. Conzentino

**Affiliations:** a Setor Litoral, UFPR Matinhos, Matinhos, Paraná, Brazil; b Campus Toledo, UFPR Toledo, Toledo, Paraná, Brazil; University of Mississippi Medical Center

**Keywords:** COVID-19, SARS-CoV-2, seroprevalence, vaccination, IgG, nucleocapsid, seroconvertion, spike

## Abstract

The investigation of antibodies raised against different severe acute respiratory syndrome coronavirus (SARS-CoV-2) antigens can help to determine the extent of previous SARS-CoV-2 infections in the population and track the humoral response to vaccination. Therefore, serological surveys can provide key information to better manage the pandemic and/or to implement the most effective vaccination program. Here we describe a time series anti-nucleocapsid, anti-spike IgG serological survey analysis in the city of Matinhos, PR, Brazil during the year of 2021. Seroconversion rates to the nucleocapsid antigen were not influenced by gender or age. The serological data support that the coronavirus disease 2019 (COVID-19) infection rate is ~50% higher than official numbers. Furthermore, by applying serological data, the corrected infection fatality rate was estimated to be lower than 2.4% in contrast with the official estimative of 3.6%. The rates of IgG reactive to spike antigen resembled the curve of the fraction the population that had taken the second vaccine dose. Up to 82% of spike seroconversion was detected in the end of 2021, confirming the effectiveness of the COVID-19 vaccination program in the city. This SARS-CoV-2 serological study unraveled the SARS-CoV-2 infection rates and the response to vaccination in the city of Matinhos.

**IMPORTANCE** The investigation of antibodies raised against SARS-CoV-2 can help to determine the extent of previous SARS-CoV-2 infections and track the humoral response to vaccination. Here we describe a time series anti-nucleocapsid, anti-spike IgG serological survey in the city of Matinhos, PR, Brazil during the year of 2021. The data depicted the progression of SARS-CoV-2 infections in the city allowing the correction of the number of citizens who experienced COVID-19 and the disease fatality rate. The seroconversion rates to the spike antigen resembled the curve of the fraction of the population that had taken the second vaccine dose, thereby confirming the effectiveness of the COVID-19 vaccination program in the city.

## INTRODUCTION

The coronavirus disease 2019 (COVID-19) pandemic has caused profound impacts in human health throughout the world. The first severe acute respiratory syndrome coronavirus (SARS-CoV-2) case was reported in Brazil by the end of February 2020. In less than 2 years, official numbers indicate that SARS-CoV-2 had infected more than 23 million, resulting in >613,000 deaths in the Brazilian territory (https://coronavirus.jhu.edu/map.html). In the state of Paraná, COVID-19 spread rapidly through all cities including Matinhos, the site of this study, where the first case was reported in March 2020. With a population of 35,705 (https://www.ibge.gov.br/cidades-e-estados/pr/matinhos.html), the city of Matinhos had reported, by November 2021, 4,052 COVID-19 cases and 147 deaths (https://www.saude.pr.gov.br/Pagina/Coronavirus-COVID-19).

Following the world spread of SARS-CoV-2, international efforts were directed toward the development of an effective vaccine. The results of these intensive global efforts came shortly by the end of 2020 when the first vaccines became available to the public. By January 2022, more than 10 billion doses were administered worldwide (https://ourworldindata.org/covid-vaccinations). In Brazil, the vaccination program started on January 2021 and more than 300 million doses were administered in 1 year. By January 2022, 63,000 vaccine doses were adminstered to the citizens of Matinhos (https://www.ibge.gov.br/cidades-e-estados/pr/matinhos.html) (total population 35,705). The COVID-19 vaccines available in Brazil were CoronaVac: Sinovac/Butantan; AstraZeneca: Oxford AZD1222 ChAdOx1; Pfizer-BioNTech: BNT162b2; and Janssen: JNJ-78436735 Ad26.COV2.S; all these vaccines have been subjected to extensive clinical trials that confirmed their safety and efficacy (1).

Serological analysis has been considered as an effective strategy to determine the prevalence of COVID-19 in the population. By tracking long-lasting IgG reactive to SARS-CoV-2 antigens, it is possible to confirm previous COVID-19 cases, which were not notified for different reasons, which may include lack of testing, wrong time of sampling to detect active infection, or unnoticed asymptomatic infections (2, 3). Serological surveys can provide important information to track the evolution of the pandemic, allowing incidence estimates at the population level. Serological analysis can also be used to track the humoral response to vaccination (2–4). The estimation of the humoral response raised by the vaccine health authorities can determine the extent of the population that has raised antibodies and for how long these antibodies are going to last. Such information is critical to optimize resources and develop an effective vaccination program. In this work, we report a year-round SARS-CoV-2 serological analysis in the city of Matinhos, Paraná, Brazil. This study provided insights into the population seroconversion in response to infection and vaccination.

## RESULTS

**FIG 1:**
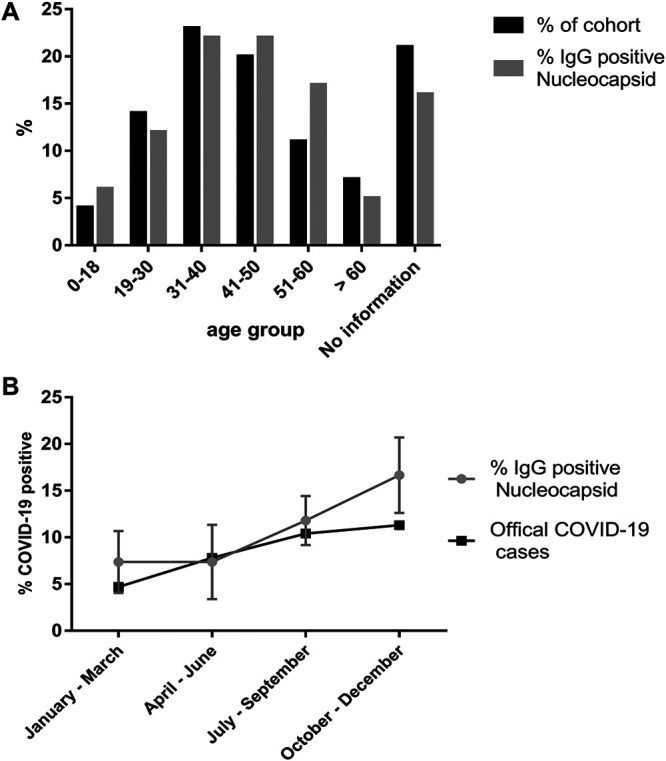
COVID-19 cases detected by IgG reactive to nucleocapsid antigen. (A) The percentage of age distribution of the cohort is shown as black bars. The percentage of IgG positive to SARS-CoV-2 nucleocapsid antigen by age group is shown as gray bars. (B) Evolution of COVID-19 cases during each trimester of 2021. The percentage of positive samples for IgG reactive to SARS-CoV-2 nucleocapsid antigen is represented by the gray line. Errors bars indicate the confidence intervals at 95% confidence level. The black line indicates the percentage of official COVID-19 cases reported by health authorities in the city of Matinhos (accumulated cases in the middle of each trimester is reported as percentage of total population).

A total of 1,384 samples were collected from 982 different individuals. All samples were analyzed for the presence of IgG reactive to SARS-CoV-2 nucleocapsid protein. Since July 2021, samples were also evaluated for the presence of IgG reacting to SARS-CoV-2 full-length spike protein (736 samples). Altogether, 2,120 serological analyses were performed. The age of the volunteers had a mean of 44 years (SD 13.5). The age distribution for the participants is presented in Fig. 1A.

Of the 1,384 samples analyzed for IgG reacting to SARS-CoV-2 nucleocapsid protein, 154 were positive, an 11.1% positive rate (95% confidence interval [CI] 9.6 to 12.6). The distribution of the positive cases by gender was similar. Women represented 64% of the cohort and 66% of the positive cases. On the other hand, men represented 36% of the cohort and 34% of the positive cases. The distribution of positive cases by age group also resembled the distribution of the cohort (Fig. 1A).

Vaccination against COVID-19 became available in Brazil by the end of January 2021. CoronaVac was among the vaccines available, representing 24.5% of the vaccines distributed in Brazil. Given that CoronaVac is based on inactivate SARS-CoV-2, it could elicit humoral response against the SARS-CoV-2 nucleocapsid antigen in such way that it would not be possible to distinguish if a nucleocapsid-positive IgG test would be caused by previous SARS-CoV-2 infection or due to CoronaVac vaccination.

To evaluate the impact of CoronaVac vaccination in our data set, we evaluated the SARS-CoV-2 nucleocapsid seroconversion rate after the exclusion of Coronavac vaccinated participants (excluded samples: *n* = 180). Quite surprisingly, the nucleocapsid IgG-positive rate was not significantly affected (11.1% versus 10.9% after exclusion). Indeed, we noticed that most subjects vaccinated with CoronaVac tested negative for IgG reactive to nucleocapsid protein. Of the 63 samples whose donors declared not to be previously diagnosed with COVID-19 and to have taken the second Coronavac dose more than 10 days, only 12% were positive (mean time between second dose and sampling: 73 days, SD 69). For all subsequent analysis considering nucleocapsid IgG data, the Coronavac vaccinated subjects were excluded. Hence, all remaining nucleocapsid IgG-positive cases would be caused only by previous SARS-CoV-2 infections. The average nucleocapsid seroconversion rate in 2021 was 10.9% (95% CI 9.6 to 12.6), while the average of official reported COVID-19 cases during 2021 in the city of Matinhos was 8.3%.

The IgG reacting to nucleocapsid seroconversion rate accordingly to the time frame of the study is depicted on Fig. 1B. The samples were categorized accordingly to the trimester of sampling during 2021. In the first trimester of the study (January to March), 7.4% of samples were positive for nucleocapsid IgG. The same number was detected in the second trimester (April to June), 11.8% in the third trimester (July to September), and finally, 16.7% in the fourth trimester (October to December). Despite the limitation of sample size in each trimester, which occured in broad confidence intervals, the data suggest that SARS-CoV-2 infections has continued to increase during the time frame of the study (Fig. 1B). The rate of nucleocapsid seroconversion followed the trend of official COVID-19 cases in the city of Matinhos (Fig. 1B). The reported official accumulated COVID-19 cases in the middle of each trimester are within the confidence intervals of the number of cases detected by nucleocapsid seroconversion, expected in the fourth trimester of 2021 (Fig. 1B).

Several studies indicate that IgG reactive to SARS-CoV-2 antigens wane over time. This reduction seems more rapidly for IgG reactive to the nucleocapsid antigen (5). The first official COVID-19 case in the city of Matinhos date back to March 30, 2020, so it would be important to understand if the seroprevalence rate for the nucleocapsid antigen could be underestimated by due to IgG waning over the time. Longitudinal data from four nucleocapsid seropositive subjects that participated in the study at least three times over >200 days indicated reduction of IgG levels in all cases. Two out of these four subjects seroreverted during the study (data not shown). Hence, the number of accumulated total COVID-19 cases in the population is likely to be underestimated in our serological survey (Fig. 1).

### Serological response to immunization.

The vaccine doses applied by October 2021 in Brazil were Pfizer-BioNTech: 37.3%; AstraZeneca: 36.6%; CoronaVac: 24.5%; and Janssen: 1.6% (https://www.gov.br/saude/pt-br/coronavirus/entregas-de-vacinas-covid-19). In order to detect the populational response to immunization, samples collected between July and November 2021 were analyzed for IgG reactive to full-length spike protein.

**FIG 2:**
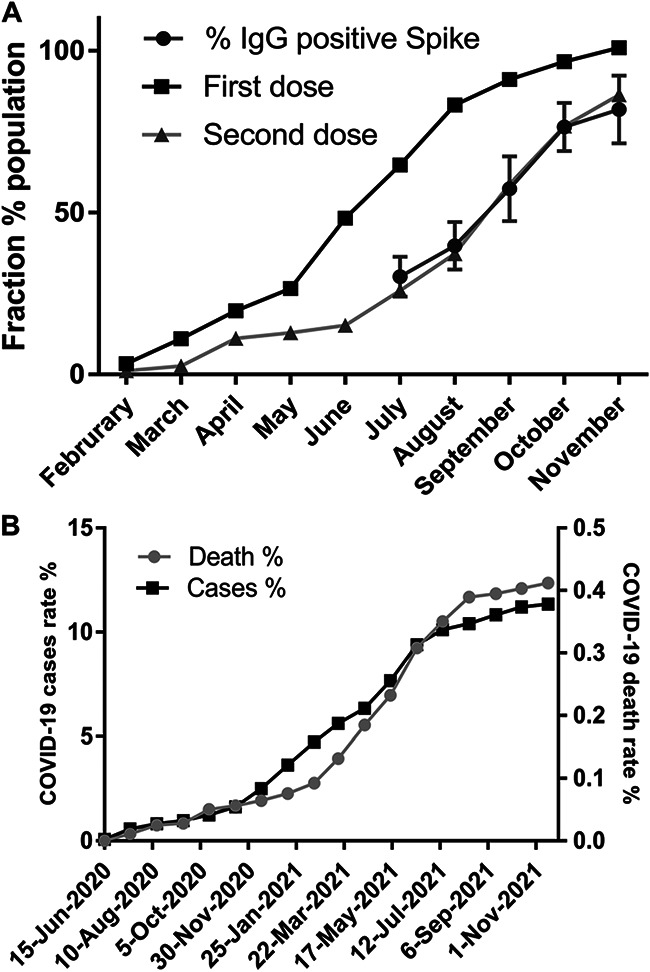
Evolution of vaccination and COVID-19 cases and deaths. (A) The fraction of the eligible (>12 years old) vaccinated population with first dose (black), with the second dose (light gray) on the Paraná State. The rate of IgG seroconversion to spike antigen detected in this study is shown in dark gray. Errors bars indicate the confidence intervals at 95% confidence level. (B) Evolution of the rate of official COVID-19 cases (black) and death (gray) in the city of Matinhos.

By July 2021, seroconversion rates were 11% to nucleocapsid and 30% to spike antigen (Fig. 1 and 2). The difference in the nucleocapsid to spike seroconversion is likely to reflect the response to vaccination given that all vaccines would elicit the humoral response to the spike antigen. The spike seroconversion rates increased sharply reacting 76% in October 2021; a plateau was apparently being reached in November 2021 when 82% spike seroconversion was detected (Fig. 2A).

It is important to mention that spike seroconversion by itself cannot distinguish between vaccinated and naturally infected individuals. Hence, the spike seroconversion rates represent the sum of both responses.

The spike seroconversion rates are well correlated with the official numbers of the second dose applied to the eligible population (>12 years old) in the Paraná state (Fig. 2A). These data clearly indicate that the vaccines available in Brazil were successful to induce the the humoral response against the spike antigen. The high level of vaccine coverage and seroconversion rates in the end of 2021 may explain the sharp decrease in COVID-19 accumulated deaths from September to December 2021 in the city of Matinhos (Fig. 2B).

It is worth mentioning that despite the full immunization with CoronaVac resulted in only 12% soroconversion for the nucleocapsid antigen as mentioned earlier, up to 47% seroconversion was detected for the spike antigen (considering samples collected >10 days after the second dose and not declared to be previously diagnostic with COVID-19; *n* = 63). For those fully immunized with the AstraZeneca vaccine, the seroconversion rate reached up to 75% >10 days after the second dose (*n* = 48). The number of samples analyzed after full immunization with Pfizer and Janssen was small (*n* < 10) and thus not considered for the rate of spike seroconversion.

## DISCUSSION

In this work, we applied serological analysis to track SARS-CoV-2 seroconversion in response to infection and vaccination during the year of 2021 in the city of Matinhos, PR, Brazil. Seroconversion for the nucleocapsid antigen was used to track previous infections after exclusion of CoronaVac vaccinated participants. On the other hand, spike antigen seroconversion was used to track the humoral response to vaccination.

Seroconversion rates to nucleocapsid antigen were not influenced by gender or age (Fig. 1A), which agrees with a previous study in Brazil (6). The nucleocapsid seroconversion rates were, in general, in agreement with official reported COVID-19 cases, especially during the second and third trimesters of 2021. More divergent numbers were observed in the first and fourth trimesters in 2021 (Fig. 1B).

The number of official cases reported in the city of Matinhos by November 2021 was 4,052. If one considers the 16.7% seroconversion rate for nucleocapsid antigen detected in November 2021, the number of total accumulated cases would be 5,962 (total population: 35,705 × 0.167). Hence, 1,910 cases were subnotified (serology corrected 5,962 to 4,052 officially reported). In other words, about 50% of the cases were subnotified (1,910 of 4,052).

It is worth mentioning that technical constrains restrict the precision of the total cases that can be estimated with the serological survey. If one considers correction for assay sensitivity (95%) and specific (99.5%), the numbers of total cases is expected to increase by a factor of 4.5%, reaching up to 6,230 cases. Furthermore, it is important to note that seroreverters were detected during this study and thus the number of real COVID-19 infections should be even higher.

The official number for the infection fatality rate (IFR) by November 2021 in Matinhos was 3.6%. If we considered the case numbers obtained by our serological survey, the IFR would be in the range of 2.4%. This number is closest to the IFR reported in Brazil and in the Paraná state of 2.7 and 2.6%, respectively (https://coronavirus.jhu.edu/map.html; https://www.saude.pr.gov.br/Pagina/Coronavirus-COVID-19), but still higher than estimated by other serological surveys in Brazil where IFRs in the range of 0.7% to 1% were estimated (6, 7). The differences in IFR numbers reported here and in other serological studies may be explained by the lack of correction for seroreversion rates. Furthermore, we speculate that local characteristics may result in higher IFR observed in the city of Matinhos; for instance, the human development index of Matinhos is below the national average and the city’s hospital can only deal with low complexity cases.

The seroconversion rates for IgG reactive to spike resembled the curve of the fraction the population that had taken the second vaccine dose (Fig. 2A). These data confirm that the vaccination campaign in Brazil was effective in activating humoral response at populational scale. The high vaccine coverage and high seroconversion rates are likely to be responsible for the reduction in official COVID-19 cases and deaths reported by the end of 2021 (Fig. 2B).

Our data unequivocally show the success of the vaccination campaign to raise antibodies against the spike antigen at the populational scale. The citizens and health authorities should be cautioned, however, because we did not investigate SARS-CoV-2-neutralizing antibodies in our analysis even though others have shown a correlation between the levels antibodies reactive to spike and neutralizing activity (8). Furthermore, longitudinal studies are lacking so we cannot predict at this stage for how long the humoral response to vaccination is going to last.

Another point to consider is the emergence of SARS-CoV-2 variants such as B.1.1.529 (Omicron), which has the potential to partially scape neutralizing antibodies raised after vaccination or after previous infections with the original SARS-CoV-2 (9). Since the first official case of B1.1.529 in the state Paraná in December 2021 (https://www.aen.pr.gov.br/Noticia/Saude-confirma-primeiro-caso-da-variante-Omicron-no-Estado), the number of official cases COVID-19 increased rapidly. Therefore, despite the high levels of seroconversion achieved by the vaccination campaign evidenced by this study we strongly recommend that heath authorities continue to stimulate social distancing and use of face masks.

In conclusion, our SARS-CoV-2 seroconversion study in the city of Matinhos helped to understand the SARS-CoV-2 infection rates and the response to vaccination. Despite the investment in molecular testing facilities, much less attention has been given by heath authorities to deploy quantitative serological analysis. Serological analyses can help to track asymptomatic cases, case contacts, and vaccine responses and act as tool to distinguish COVID-19 from other diseases causing related symptoms such as dengue fever and influenza. We suggest that the implementation of SARS-CoV-2 serological analysis as part of the public health services would provide key information to better manage the COVID-19 pandemic.

## MATERIALS AND METHODS

### Study design and sampling.

A local COVID-19 seroprevalence survey was conducted between January and December 2021 in the city of Matinhos, Paraná, Brazil. Participants were enrolled for the study by advertisement at university web site, radio, and television. Ethics approval was obtained from the CEP/UFPR (no. 35872520.8.0000.0102). Informed consent was obtained from all participants. A questionnaire was presented online (the questionnaire was optional in the beginning of the study but became mandatory since June 2021). In the questionnaire, participants provided personal information: age, gender, and the city of residence. We initially attempted to add a self-declaration for the participant to inform a previous quantitative RT-qPCR COVID-19-positive diagnosis. However, as different types of COVID-19 tests became available during this study (antigen and serological), most participants were not able to distinguish between the different types of tests. Therefore, a simpler form was used in which the participant self-declared a previous COVID-19 diagnosis and provided the date of symptom onset. During this study, vaccination against COVID-19 became available so we included a self-declaration questionnaire for COVID-19 immunization, which became mandatory to fill out since June 2021. Participants had to indicate if they had been vaccinated, the vaccine manufacturer’s popular name (CoronaVac, AstraZeneca, Pfizer, or Janssen), and the date of the first, second, and third doses (if any).

Sampling campaigns were performed at Federal University of Paraná in the city of Matinhos. Approximately 0.1 mL of blood was collected by finger puncture. Samples were centrifuged (5,000 × *g* for 3 min), and four microliters serum was used to investigate IgG reactive against the SARS-CoV-2 nucleocapsid or full prefusion spike using an in-house high-throughput magnetic immunofluorescent assay, which has been described previously (10, 11). The details of antigen purification and bead preparation have been described previously (12, 13). The results of each sample were reported and the percentage of a reference serum. The method operated with specificity and sensitivity of >99.5% and >95%, respectively (10).

Confidence intervals were calculated using the online application (https://www.surveysystem.com/sscalc.htm). The population of the city of Matinhos was estimated in 35,705 (https://www.ibge.gov.br/cidades-e-estados/pr/matinhos.html), and prevalence values of 10% or 50% were used to obtain confidence intervals for nucleocapsid and spike IgG analysis, respectively. The seroprevalence results were compared with official numbers of reported COVID-19 cases and deaths occurring in the city of Matinhos and with the official numbers of applied vaccines in the state of Paraná. These data are available from the Paraná State Secretary of Health (https://www.saude.pr.gov.br/Pagina/Coronavirus-COVID-19). The estimated population of the Paraná state was 11,597,484, and the vaccine eligible population (>12 years old) in 2021 was estimated to be 8,883,672 based on the population pyramid predictions by the Brazilian Institute of Geography and Statistics IBGE (https://www.ibge.gov.br/apps/populacao/projecao/box_piramideplay.php?ag=41).

### Data availability.

The data that support the findings of this study are available on request from the corresponding author (L.F.H.).

## References

[1] Kim JH, Marks F, Clemens JD. 2021. Looking beyond COVID-19 vaccine phase 3 trials. Nat Med 27:205–211. doi:10.1038/s41591-021-01230-y. 33469205

[2] Huang AT, Garcia-Carreras B, Hitchings MDT, Yang B, Katzelnick LC, Rattigan SM, Borgert BA, Moreno CA, Solomon BD, Trimmer-Smith L, Etienne V, Rodriguez-Barraquer I, Lessler J, Salje H, Burke DS, Wesolowski A, Cummings DAT. 2020. A systematic review of antibody mediated immunity to coronaviruses: kinetics, correlates of protection, and association with severity. Nat Commun 11:4704. doi:10.1038/s41467-020-18450-4. 32943637 PMC7499300

[3] Petherick A. 2020. Developing antibody tests for SARS-CoV-2. Lancet 395:1101–1102. doi:10.1016/S0140-6736(20)30788-1. 32247384 PMC7270070

[4] Long Q-X, Tang X-J, Shi Q-L, Li Q, Deng H-J, Yuan J, Hu J-L, Xu W, Zhang Y, Lv F-J, Su K, Zhang F, Gong J, Wu B, Liu X-M, Li J-J, Qiu J-F, Chen J, Huang A-L. 2020. Clinical and immunological assessment of asymptomatic SARS-CoV-2 infections. Nat Med 26:1200–1204. doi:10.1038/s41591-020-0965-6. 32555424

[5] Ortega N, Ribes M, Vidal M, Rubio R, Aguilar R, Williams S, Barrios D, Alonso S, Hernández-Luis P, Mitchell RA, Jairoce C, Cruz A, Jimenez A, Santano R, Méndez S, Lamoglia M, Rosell N, Llupià A, Puyol L, Chi J, Melero NR, Parras D, Serra P, Pradenas E, Trinité B, Blanco J, Mayor A, Barroso S, Varela P, Vilella A, Trilla A, Santamaria P, Carolis C, Tortajada M, Izquierdo L, Angulo A, Engel P, García-Basteiro AL, Moncunill G, Dobaño C. 2021. Seven-month kinetics of SARS-CoV-2 antibodies and role of pre-existing antibodies to human coronaviruses. Nat Commun 12:4740. doi:10.1038/s41467-021-24979-9. 34362897 PMC8346582

[6] Hallal PC, Hartwig FP, Horta BL, Silveira MF, Struchiner CJ, Vidaletti LP, Neumann NA, Pellanda LC, Dellagostin OA, Burattini MN, Victora GD, Menezes AMB, Barros FC, Barros AJD, Victora CG. 2020. SARS-CoV-2 antibody prevalence in Brazil: results from two successive nationwide serological household surveys. Lancet Glob Health 8:e1390–1398–e1398. doi:10.1016/S2214-109X(20)30387-9. 32979314 PMC7511212

[7] Marra V, Quartin M. 2021. A Bayesian estimate of the early COVID-19 infection fatality ratio in Brazil based on a random seroprevalence survey. Int J Infect Dis 111:190–195. doi:10.1016/j.ijid.2021.08.016. 34390858 PMC8358085

[8] Ng DL, Goldgof GM, Shy BR, Levine AG, Balcerek J, Bapat SP, Prostko J, Rodgers M, Coller K, Pearce S, Franz S, Du L, Stone M, Pillai SK, Sotomayor-Gonzalez A, Servellita V, Martin CSS, Granados A, Glasner DR, Han LM, Truong K, Akagi N, Nguyen DN, Neumann NM, Qazi D, Hsu E, Gu W, Santos YA, Custer B, Green V, Williamson P, Hills NK, Lu CM, Whitman JD, Stramer SL, Wang C, Reyes K, Hakim JMC, Sujishi K, Alazzeh F, Pham L, Thornborrow E, Oon C-Y, Miller S, Kurtz T, Simmons G, Hackett J, Busch MP, Chiu CY. 2020. SARS-CoV-2 seroprevalence and neutralizing activity in donor and pantient blood. Nat Commun 11:4698. doi:10.1038/s41467-020-18468-8. 32943630 PMC7499171

[9] Planas D, Saunders N, Maes P, Guivel-Benhassine F, Planchais C, Buchrieser J, Bolland W-H, Porrot F, Staropoli I, Lemoine F, Péré H, Veyer D, Puech J, Rodary J, Baele G, Dellicour S, Raymenants J, Gorissen S, Geenen C, Vanmechelen B, Wawina-Bokalanga T, Martí-Carreras J, Cuypers L, Sève A, Hocqueloux L, Prazuck T, Rey FA, Simon-Loriere E, Bruel T, Mouquet H, André E, Schwartz O. 2022. Considerable escape of SARS-CoV-2 Omicron to antibody neutralization. Nature 602:671–675. doi:10.1038/s41586-021-04389-z. 35016199

[10] Conzentino MS, Santos TPC, Selim KA, Wagner B, Alford JT, Deobald N, Paula NM, Rego FGM, Zanette DL, Aoki MN, Nardin JM, Huergo MCC, Reis RA, Huergo LF. 2021. Ultra-fast, high throughput and inexpensive detection of SARS-CoV-2 seroconversion using Ni^2+^ magnetic beads. Anal Biochem 631:114360. doi:10.1016/j.ab.2021.114360. 34481802 PMC8413102

[11] Huergo LF, Selim KA, Conzentino MS, Gerhardt ECM, Santos ARS, Wagner B, Alford JT, Deobald N, Pedrosa FO, de Souza EM, Nogueira MB, Raboni SM, Souto D, Rego FGM, Zanette DL, Aoki MN, Nardin JM, Fornazari B, Morales HMP, Borges VA, Nelde A, Walz JS, Becker M, Schneiderhan-Marra N, Rothbauer U, Reis RA, Forchhammer K. 2021. Magnetic bead-based immunoassay allows rapid, inexpensive, and quantitative detection of human SARS-CoV-2 antibodies. ACS Sens 6:703–708. doi:10.1021/acssensors.0c02544. 33496577

[12] Alvim RGF, Lima TM, Rodrigues DAS, Marsili FF, Bozza VBT, Higa LM, Monteiro FL, Leitão IC, Carvalho RS, Galliez RM, Castineiras TM, Nobrega A, Travassos LH, Ferreira OC, Jr, Tanuri A, Vale AM, Castilho LR. 2020. An affordable anti-SARS-COV-2 spike protein ELISA test for early detection of IgG seroconversion suited for large-scale surveillance studies in low-income countries. medRxiv. doi:10.1101/2020.07.13.20152884.

[13] Conzentino MS, Forchhammer K, Souza EM, Pedrosa FO, Nogueira MB, Raboni SM, Rego FGM, Zanette DL, Aoki MN, Nardin JM, Fornazari B, Morales HMP, Celedon PAF, Lima CVP, Mattar SB, Lin VH, Morello LG, Marchini FK, Reis RA, Huergo LF. 2021. Antigen production and development of an indirect ELISA based on the nucleocapsid protein to detect human SARS-CoV-2 seroconversion. Braz J Microbiol 52:2069–2073. doi:10.1007/s42770-021-00556-6. 34342836 PMC8329412

